# A dimension reduction technique applied to regression on high dimension, low sample size neurophysiological data sets

**DOI:** 10.1186/s12868-020-00605-0

**Published:** 2021-01-04

**Authors:** Adrielle C. Santana, Adriano V. Barbosa, Hani C. Yehia, Rafael Laboissière

**Affiliations:** 1 Graduate Program in Electrical Engineering, Universidade Federal de Minas Gerais, Av. Pres. Antônio Carlos 6627, 31270-901 Belo Horizonte, Brazil; 2 Univ. Grenoble Alpes, CNRS, LPNC UMR 5105, 38000 Grenoble, France; 3 Department of Control and Automation Engineering, School of Mines, Universidade Federal de Ouro Preto, Campus Morro do Cruzeiro, 35400-000 Ouro Preto, Brazil; 4 Department of Electronic Engineering, Universidade Federal de Minas Gerais, Av. Pres. Antônio Carlos 6627, 31270-901 Belo Horizonte, Brazil

**Keywords:** Electroencephalography, Event-related potentials, Linear regression, High dimension low sample size problem, Dimension reduction, Phonemic categorization, Discrete wavelet transform

## Abstract

**Background:**

A common problem in neurophysiological signal processing is the extraction of meaningful information from high dimension, low sample size data (HDLSS). We present RoLDSIS (regression on low-dimension spanned input space), a regression technique based on dimensionality reduction that constrains the solution to the subspace spanned by the available observations. This avoids regularization parameters in the regression procedure, as needed in shrinkage regression methods.

**Results:**

We applied RoLDSIS to the EEG data collected in a phonemic identification experiment. In the experiment, morphed syllables in the continuum /da/–/ta/ were presented as acoustic stimuli to the participants and the event-related potentials (ERP) were recorded and then represented as a set of features in the time-frequency domain via the discrete wavelet transform. Each set of stimuli was chosen from a preliminary identification task executed by the participant. Physical and psychophysical attributes were associated to each stimulus. RoLDSIS was then used to infer the neurophysiological axes, in the feature space, associated with each attribute. We show that these axes can be reliably estimated and that their separation is correlated with the individual strength of phonemic categorization. The results provided by RoLDSIS are interpretable in the time-frequency domain and may be used to infer the neurophysiological correlates of phonemic categorization. A comparison with commonly used regularized regression techniques was carried out by cross-validation.

**Conclusion:**

The prediction errors obtained by RoLDSIS are comparable to those obtained with Ridge Regression and smaller than those obtained with LASSO and SPLS. However, RoLDSIS achieves this without the need for cross-validation, a procedure that requires the extraction of a large amount of observations from the data and, consequently, a decreased signal-to-noise ratio when averaging trials. We show that, even though RoLDSIS is a simple technique, it is suitable for the processing and interpretation of neurophysiological signals.

## Background

Functional brain imaging experiments are currently used in studies that aim to identify the neurophysiological correlates of perception. In these experiments, it is assumed that a given perceptual stimulus evokes a specific pattern of neuronal activity in the central nervous system. This activity can be captured through a variety of measurements, like electric potentials in electroencephalography (EEG) and electrocorticography (ECoG), magnetic fields in magnetoencephalography (MEG), blood flow changes in near infrared spectroscopy (NIRS), or haemodynamic response in functional magnetic resonance imaging (fMRI). The recorded signals are usually represented in time and frequency (through spectro-temporal analysis, like Fourier or wavelet transforms), as well as in the physical space (EEG or MEG sensors, or fMRI voxels).

These measurements represent the evoked response in the brain and can be mathematically represented as vectors in an ${\mathbb {R}}^N$ space, where *N* is the total number of *features* used to represent the EEG measurements. Each feature corresponds to a discrete point in time, frequency, and spatial domains. The dimension of this representation space is usually very high. For instance, consider an EEG experiment with 64 electrodes in which the event-related potential (ERP) lasts for 0.5 s and is represented in the time-frequency domain by a spectrogram with ten binned frequency bands and sampled in time every 1 ms. This would result in a representation space containing $64 \times 500$ $\times$ 10 = 320,000 features. Such high dimensions are not uncommon in brain imaging studies.

In EEG experiments, the ERP evoked by the stimulus corresponds to electric potential fluctuations which are very small in comparison with the ongoing, background electric activity measured on the scalp. In order to obtain reliable measures of the ERP for each stimulus, it is necessary to average the responses ss a large amount of trials. Depending on the desired signal-to-noise ratio (SNR), several hundreds, or sometimes thousands of trials are required to obtain reliable ERPs [1]. This requirement imposed by the SNR is also critical in other cases, such as in studies of epileptic seizures, in which measurements may take up days in order to detect epileptogenic zones [2, 3], and in brain-computer interface (BCI) systems that rely on a small amount of EEG observations for inferring the intention of the user [4, 5]. At any rate, due to time limitations in recording the data for a single participant, typical EEG experiments involve a limited amount of *observations*, which are the ERPs for each stimulus.

In the present paper, we are interested in the neurophysiological correlates of perception, in the context of EEG experiments involving a small amount of observations. We assume that each stimulus *i* used in the experiment can be characterized by a scalar *attribute* $y_i \in {\mathbb {R}}$. We also assume that the attribute $y_i$ has a functional relationship with the observations (represented here by the vectors ${\mathbf {x}}_i$), expressed as $y = f({\mathbf {x}})$. Here, we consider the simplest, linear approximation for this relationship, the affine transformation:

1 $$\begin{aligned} y = a + {\mathbf {b}}^\intercal {\mathbf {x}}. \end{aligned}$$

The vector ${\mathbf {b}} \in {\mathbb {R}}^N$ represents the *neurophysiological axis* related to the attribute *y*. The neurophysiological axis determines how the features in ${\mathbf {x}}$ must be combined in order to yield the value associated with the stimulus attribute *y*. The vector ${\mathbf {b}}$ and the scalar *a* must be inferred from the available *M* pairs observations/attributes $\{{\mathbf {x}}_i,y_i\}$.

Since the affine transformation is only an approximation to the true relationship between the domains, the *M* observations are related through the equation:

2 $$\begin{aligned} y_i = a + {\mathbf {b}}^\intercal {\mathbf {x}}_i + \epsilon _i, i = 1,\ldots ,M, \end{aligned}$$

where $\epsilon _i$ are assumed to be independent and identically distributed random errors that follow the normal distribution. This is a regression problem which can be solved by minimizing the quadratic error function:

3 $$\begin{aligned} E(a, {\mathbf {b}} | \{{\mathbf {x}}_i, y_i\}) = \sum _{i=1}^M \epsilon _i^2 = \sum _{i=1}^M \left( y_i - a - {\mathbf {b}}^\intercal {\mathbf {x}}_i\right) ^ 2. \end{aligned}$$

When $M < N$, the problem is said to be underdetermined, meaning that there is an infinite number of values of *a* and ${\mathbf {b}}$ that yield an exact solution. In the case of EEG experiments, as we described above, this problem is exacerbated because *N* is much larger than *M*. This results in the so-called high dimension, low sample size (HDLSS) problem [6, 7]. Indeed, the data set is very sparse in a space represented by a high number of features, many of them being potentially irrelevant or redundant for describing the underlying neuronal processes. This phenomenon has been called “the curse of dimensionality” [8].

Techniques of regularization or variable selection, such as least absolute shrinkage and selection operator (LASSO), Ridge Regression [9], and sparse partial least squares (SPLS) [10], can be used to obtain a well-posed optimization problem expressed by Eq. 3, which is formulated as:

4 $$\begin{aligned} \min _{\{a, {\mathbf {b}}\}} \left[ E(a, {\mathbf {b}} | \{{\mathbf {x}}_i, y_i\}) + \lambda P({\mathbf {b}})\right] , \end{aligned}$$

where $\lambda$ is a regularization parameter and *P* is a penalty function for the regression coefficients in vector ${\mathbf {b}}$. In general, the parameter $\lambda$ cannot be determined *a priori* and must be inferred from the data, using some kind of cross-validation (CV) procedure. This is possible when there is an abundant number of pairs $\{{\mathbf {x}}_i, y_i\}$ in order to feed the CV procedure.

Here, we propose an alternative regression technique, which is a specific case of the dimension reduction methods described in [11] that avoids the problem of specifying regularization parameters when the number of observations is very small. We call it regression on low-dimension spanned input space (RoLDSIS).

## Methods

The main idea behind the RoLDSIS technique is to assume that the neurophysiological axis ${\mathbf {b}}$ is restricted to the $(M-1)$-dimensional linear subspace spanned by the *M* linearly independent vectors ${\mathbf {x}}_i$, which are the only available observations:

5 $$\begin{aligned} {\mathbf {x}}_i = [x_{i1}\ x_{i2} \ldots x_{iN}]^\intercal ,\quad i = 1,\ldots ,M, \end{aligned}$$

where *N* is the number of representation features and $^\intercal$ denotes transpose.

The $(M-1)$-dimensional subspace spanned by the observations can be obtained by principal component analysis (PCA) applied to the *M* vectors ${\mathbf {x}}_i$. Since the dimension *N* of the feature space is greater than the number of observations *M*, the PCA yields $M-1$ normalized eigenvectors, which define a basis for the spanned subspace.

Let ${\mathbf {V}}$ be the matrix whose columns are the PCA eigenvectors. We can obtain the projections of ${\mathbf {x}}$ onto this $(M-1)$-dimensional spanned subspace by making

6 $$\begin{aligned} {\mathbf {z}} = {\mathbf {V}}^\intercal ({\mathbf {x}} - {\mathbf {m}}), \end{aligned}$$

where ${\mathbf {m}}$ is the mean of the observations ${\mathbf {x}}_i$.

For the particular case where ${\mathbf {x}}$ is contained in the spanned subspace, we have

7 $$\begin{aligned} {\mathbf {x}} = {\mathbf {V}} {\mathbf {z}} + {\mathbf {m}}. \end{aligned}$$

Thus, if we restrict the solutions of Eq. 1 to the spanned subspace, it can be expressed as

8 $$\begin{aligned} y&= a + {\mathbf {b}}^\intercal ({\mathbf {V}} {\mathbf {z}} + {\mathbf {m}}), \end{aligned}$$

9 $$\begin{aligned}&= a + {\mathbf {b}}^\intercal {\mathbf {m}} + {\mathbf {b}}^\intercal {\mathbf {V}} {\mathbf {z}}. \end{aligned}$$

By making $c = a + {\mathbf {b}}^\intercal {\mathbf {m}}$ and ${\mathbf {d}} = {\mathbf {V}}^\intercal {\mathbf {b}}$, Eq. 9 becomes,

10 $$\begin{aligned} y = c + {\mathbf {d}}^\intercal {\mathbf {z}}, \end{aligned}$$

which has *M* unknowns: the scalar *c* and the $(M-1)$ components of the vector ${\mathbf {d}}$. The *M* pairs $\{{\mathbf {x}}_i, y_i\}$ can now be used to define the linear system with *M* unknowns and *M* equations

11 $$\begin{aligned} y_i = c + {\mathbf {d}}^\intercal {\mathbf {z}}_i, \quad i = 1,\ldots ,M. \end{aligned}$$

Since we assumed that the observations are linearly independent, this is an even-determined problem. There is only one solution that satisfies the equations exactly:

12 $$\begin{aligned} {\mathbf {d}}&= {\mathbf {V}}^\intercal {\mathbf {b}}, \end{aligned}$$

13 $$\begin{aligned} c&= a + {\mathbf {b}}^\intercal {\mathbf {m}}, \end{aligned}$$

yielding

14 $$\begin{aligned} {\mathbf {b}}&= {\mathbf {V}} {\mathbf {d}}, \end{aligned}$$

15 $$\begin{aligned} a&= c - {\mathbf {d}}^\intercal {\mathbf {V}}^\intercal {\mathbf {m}}. \end{aligned}$$

Finally, the original observations ${\mathbf {x}}_i$ can be projected onto the normalized neurophysiological axis $\hat{{\mathbf {b}}} = {\mathbf {b}} / ||{\mathbf {b}}||$, yielding the representations

16 $$\begin{aligned} \tilde{{\mathbf {x}}}_i = {\mathbf {m}} + \hat{{\mathbf {b}}} [\hat{{\mathbf {b}}}^\intercal (\mathbf {x_i} - {\mathbf {m}})], \end{aligned}$$

which can be used to infer the underlying brain states related to the stimuli attributes $y_i$.

**Fig. 1:**
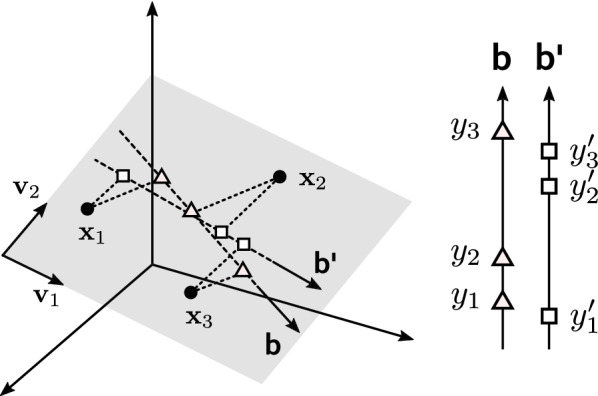
Graphic representation of the RoLDSIS technique. See the Methods section for details

A geometric representation of the RoLDSIS technique is illustrated in Fig. 1, for the case of $M=3$ ${\mathbf {x}}_i$ observations contained in a feature space of dimension $N=3$. These three observations, represented as black dots, span a subspace of dimension $N-1=2$, which is depicted by the gray plane in the figure. This subspace is defined by the two orthonormal vectors ${\mathbf {v}}_1$ and ${\mathbf {v}}_2$. Notice that, for the sake of visual clarity, the origin of the coordinate system $\{{\mathbf {v}}_1,{\mathbf {v}}_2\}$ is displaced to the border of the quadrilateral representing the plane, instead of being at the mean point ${\mathbf {m}}$ (see Eq. 6). In the figure, two hypothetical neurophysiological axes, denoted by ${\mathbf {b}}$ and $\mathbf {b'}$, are also depicted. These axes, which are contained in the bi-dimensional subspace, are related to two different stimuli attributes *y* and $y'$. The projections of the three observations ${\mathbf {x}}_i$ onto the ${\mathbf {b}}$ and the $\mathbf {b'}$ axes are represented by triangles and squares, respectively. On the right side of the figure, these axes are shown again, with the respective scalar values of the attributes $y_i$ and $y'_i$ associated with the projected observations. In this specific example, one can see how the same observations ${\mathbf {x}}_i$ can have qualitatively different interpretations along the two different neurophysiological axes. For the ${\mathbf {b}}$ axis, stimulus #2 is closer to stimulus #1 than to stimulus #3, while the converse happens for the $\mathbf {b'}$ axis.

## Results

### Example data: neurophysiological correlates of phonemic identification

We validated the RoLDSIS technique using data from an experiment that addressed *categorical perception*. Perceptual categorization involves neuronal mechanisms accounting for the transformation of lower-level sensory inputs, which capture the continuous properties of the stimulus, into a higher-level conceptual representation, which is composed of discrete classes or categories. This is the case, for example, of the perception of colors and facial emotions [12]. Categorical perception also happens in speech, where sounds with continuous physical attributes are mapped onto discrete perceptual classes, in a process called phonemic categorization [13].

Over the past decades, categorical perception in speech has been studied from both the behavioral and theoretical points of view [14]. More recently, improvements in technologies for brain activity measurement, as well as the availability of computational power, have allowed the investigation of the neurophysiological mechanisms underlying this phenomenon. Using direct electrode recordings of patients undergoing preoperative surgery, Chang and colleagues [15] recorded the cortical responses to the phonemic continuum /ba/-/da/-/ga/ in the secondary auditory cortex. By applying pattern recognition techniques, they showed that neuronal activity mirrors perception, demonstrating that categorical representation arises around 110 ms after stimulus onset. Using a less invasive acquisition system (EEG), Bidelman and colleagues [16] investigated the emergence of categorical perception for the phonemic continuum /u/-/a/. By analyzing the frequency band involved in the N1–P2 complex at the temporal scalp region, they showed that the neurophysiological correlates of phonemic categorization emerges around 175 ms after stimulus onset. They also showed that physical properties of the stimulus (vowel formants, in their case) are encoded in the early, high-frequency bands of the auditory response, probably coming from sub-cortical regions. Using fMRI and MEG during syllable identification, Bouton and colleagues [17] showed that activities related to sensory and categorization processing happens in a restricted part of the posterior superior temporal gyrus. They also showed that neuronal activity in this region reflect the syllable identification errors.

These studies indicate that it is possible to investigate the neurophysiological correlates of phonemic categorization. However, none of them tried to infer the neuronal representation of both the physical ($\phi$) and psychophysical ($\psi$) attributes of the stimuli directly from the full set of features available in the evoked auditory responses. This is a situation that is particularly well suited for the application of the RoLDSIS procedure. In the subsections that follow, we describe a phonemic identification experiment using EEG, whose data will be used to validate the new regression technique proposed in the present paper.

#### Participants

Eleven participants, five males and six females, aged 28 years on average (SD 9 years), participated voluntarily in the experiment. All participants were native speakers of Brazilian Portuguese. They were all right-handed and presented an average grade of 76.8 for the right hand, according to Oldfield’s laterality index [18]. None of them had any history of neurological, language, or auditory disorders. All participants were previously informed about the procedures and tasks of the experiment and provided written informed consent to participate in the study. The experiment was approved by the Ethics Committee of the Federal University of Minas Gerais (COEP-UFMG, Brazil).

#### Auditory stimuli

A continuum of sounds was created between the syllables /da/ and /ta/, which were recorded from a male speaker of Brazilian Portuguese, who uttered both syllables in isolation in a natural setting inside a sound-proof booth. Both syllables finish with the open front unrounded vowel /a/. The initial consonants are the alveolar stops /t/ and /d/. These consonants have the same point of articulation at the front of the vocal tract, but differ in voice onset time (VOT), which is the amount of time between the occlusion release and the voicing arising from the vibration of the vocal folds (negative for /da/ and positive for /ta/) [19]. A morphing procedure was used to generate the 200 intermediary, synthetic stimuli of the continuum. These stimuli were created by continuously varying the onset of the voicing murmur of /da/, from −52 ms to 0 ms. In all cases, the release burst is present, resulting in a $+$16 ms VOT value for the extreme /ta/ syllable. Each stimulus was saved to an audio WAV file. The reference time was chosen to be the beginning of the stationary part of the vowel, such that the beginning of the WAV file corresponds to $t=-$74 ms in the original stimuli. Thus, the stationary part of the vowel for all stimuli was temporally aligned, in relation to the beginning of the WAV file. The duration of the WAV files is 220 ms.

#### Identification task

**Fig. 2:**
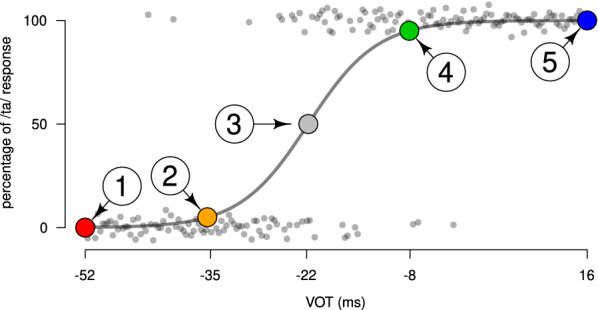
Results of the phonemic identification task for a representative participant. Responses to the 200 stimuli, each one for a specific value of voice onset time (along the horizontal axis) are shown as gray dots around 0.0 (for /da/ responses) and around 1.0 (for /ta/ responses). Vertical jitter has been added for the sake of clarity. The gray curve is the theoretical psychometric response fitted to the data. Choices of stimuli #1, #2, #3, #4, and #5, corresponding to 0%, 5%, 50%, 95% and 100% of /ta/ responses, respectively, are shown by colored dots on the psychometric curve. The VOT values for the stimuli are indicated in the horizontal axis

The participants were tested in a preliminary phonemic identification experiment, in which the stimuli were presented in random order through earphones. The participant’s task was to identify the perceived syllable (/da/ or /ta/) in a forced binary choice task. The results of this experiment are shown in are Fig. 2 for a representative participant, where a psychometric, logistic curve was fitted to the participant’s responses using the glmrob function of the R software [20]. In this figure, the values 0% and 100% correspond, respectively, to the identification of the /da/ and /ta/ syllables. For the subsequent EEG experiment, the stimuli corresponding to 0%, 5%, 50%, 95%, and 100% of the psychometric curve were selected. Hereafter, these stimuli are called #1, #2, #3, #4, and #5, respectively. Stimuli #2 and #4 are closer to stimulus #3 from the acoustic (physical) point of view (abscissa axis), and closer to the extreme stimuli #1 and #5, respectively, from the perceptual (psychophysical) point of view (ordinate axis).

#### EEG experiment

**Fig. 3:**
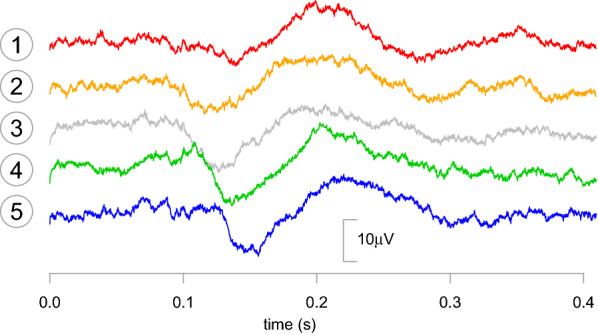
Event-related potentials for a representative participant (the same as in Fig. 2). Averaged ERPs for stimuli #1, #2, #3, #4, and #5 are shown from top to bottom. Only the 128 wavelet components in the low frequency bands (below 156 Hz) are considered for the signal reconstruction. Due to the baseline correction, all signals are close to zero at $t =$ 0 s. The signals are displaced vertically for the sake of visualization

Each participant was subsequently tested in an EEG experiment, where the five selected stimuli were presented in random order, 200 times each. The participant was asked to perform the same phonemic identification task as done previously. We recorded the activity of the electrodes placed at the vertex of the head and on the mastoid bone, behind the left ear. These are the placement of electrodes typically used in the study of speech processing in the central nervous system, including the auditory brainstem response (ABR) and the responses in the auditory cortex (temporal lobe) [21]. This choice of placement of the electrodes should then allow the recording of the underlying neuronal activity used both in phonemic feature processing and categorization [15, 22]. The electric potentials between these two electrodes were acquired with passive gold cup electrodes connected to an RHD2000 acquisition board (Intan Technologies) and sampled at 5 kHz. The signal was epoched by stimulus response and time-locked to the onset of signal in the WAV file. The response in each trial was baseline corrected using a 150 ms pre-stimulus time. Excessively noisy trials were removed by visual inspection, what resulted in around 91.5% of the trials being kept for subsequent analysis. Each trial has 2048 time samples (lasting for around 0.4 s). Figure 3 illustrates the ERPs for each stimulus for a representative participant. The SNR of the raw signal was estimated from the ERP to be between − 12 and − 15 dB.

#### Feature extraction

Discrete wavelet transform (DWT) was applied to each trial, in order to obtain its representation in the time-frequency domain [23]. The DWT is obtained by filtering the signal through a series of high and low-pass filters in a recursive filtering and downsampling process. Since the DWT is a linear and orthogonal transformation, it provides a parsimonious representation of the signal being transformed, with each resulting coefficient associated with a specific location in the time-frequency domain [24]. Furthermore, the DWT performs the decomposition of the signal into frequency bands, what is useful for the analysis of rhythmic patterns of neuronal activity in the central nervous system. In our case, the DWT yields a set of 2048 coefficients, organized in blocks. The first block contains the so-called *approximation coefficients* (V) and comprise a low-pass filtered representation of the signal. The remaining blocks contain the *detail coefficients* (W), which comprises the high frequency information. These coefficients are obtained by convolving the signal with a band-pass filter based on the *mother wavelet* [25]. We used as mother wavelet the Daubechies orthonormal compactly supported wavelet of length 8, from the least asymmetric family, available in the package wavelets of the R software [26]. Only the DWT coefficients corresponding to the low frequency bands (between 0 and 156 Hz) were retained, resulting in a feature vector of length 128. This range of frequencies covers the bands $\uptheta$, $\upalpha$, $\upbeta$, and $\upgamma$, which are of interest in brain electrophysiology studies of speech perception [27, 28]. The feature vectors were averaged across trials for each participant and each stimulus. The RoLDSIS technique was then applied to these averaged observations ${\mathbf {x}}_i, i = 1,\ldots ,5$.

### Application of the RoLDSIS technique

#### Linear regression on physical and psychophysical attributes

As explained above, each stimulus *i* is associated with both a specific physical attribute $\phi _i$ (VOT value of the associated stimulus) and with a specific psychophysical attribute $\psi _i$ (the proportion of /ta/ responses of the associated stimulus, obtained from the psychometric curve). For the psychophysical attribute, we used the proportions of /ta/ responses corresponding to the selected stimuli $\psi _1 = 0.0$, $\psi _2 = 0.05$, $\psi _3 = 0.5$, $\psi _4 = 0.95$, and $\psi _5 = 1.0$. For the physical attributes, we used the VOT of the selected stimuli. The first ($\phi _1$) and last ($\phi _5$) values were equal for all participants and corresponded to the /da/ and /ta/ stimuli at the beginning and at the end of the continuum (−52 ms and $+$16 ms, respectively). The other three values varied for each participant, since the psychometric curve is idiosyncratic. For instance, for the participant whose psychometric curve is depicted in Fig. 2, the physical attributes were $\phi _2 = -35$ ms, $\phi _3 = -22$ ms, and $\phi _4 = -8$ ms.

We hypothesize the following linear relationships, for $i = 1,\ldots ,5$:

17 $$\begin{aligned} \phi _i&= a_\Phi + {\mathbf {b}}_\Phi ^\intercal {\mathbf {x}}_i, \end{aligned}$$

18 $$\begin{aligned} \psi _i&= a_\Psi + {\mathbf {b}}_\Psi ^\intercal {\mathbf {x}}_i, \end{aligned}$$

where ${\mathbf {x}}_i, \; i = 1,\ldots ,5$, are the observation vectors obtained from the DWT analysis described above. We assume that ${\mathbf {b}}_\Phi$ and ${\mathbf {b}}_\Psi$ are unit vectors (*ie* $||{\mathbf {b}}|| = 1$). Since ${\mathbf {x}}_i \in {\mathbb {R}}^{128}$, vectors ${\mathbf {b}}_\Phi$ and ${\mathbf {b}}_\Psi$ have 127 free coefficients to be determined. Considering also the scalar parameters $a_\Phi$ and $a_\Psi$, each equation above results in a system of 5 linear equations with 128 unknowns.

**Fig. 4:**
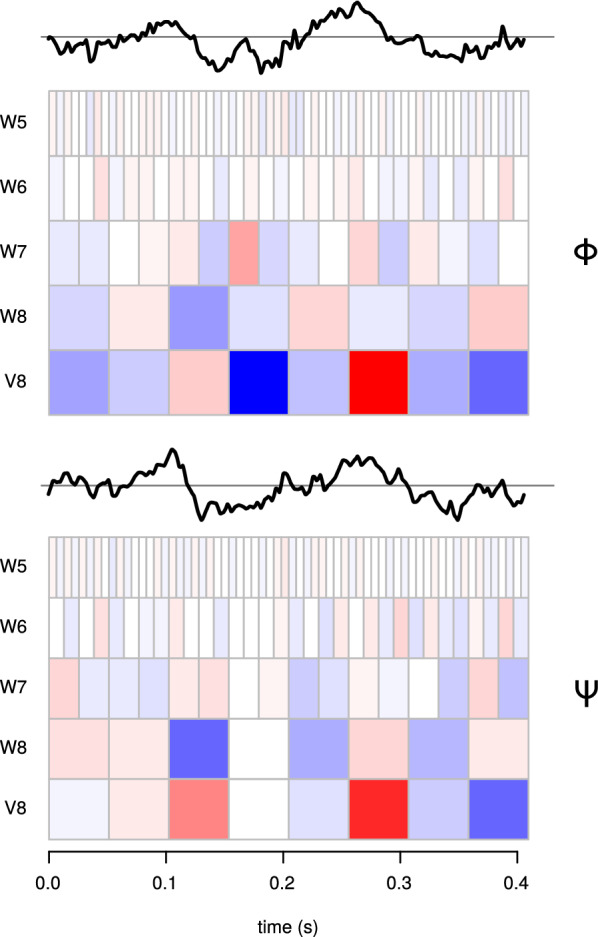
Direction obtained for the RoLDSIS procedure for a representative participant (the same as in Fig. 2. The results of the RoLDISS for the physical ($\Phi$) and psychophysical ($\Psi$) observations are shown in the top and bottom panels, respectively. In each panel, the time-domain representation of the optimal direction vector, obtained by applying the inverse DWT on the RoLDSIS result is shown by the black line, which is atop of the scalogram (time/frequency representation) of this direction vector. The amplitudes of the DWT coefficients are represented in a color scale, negative values in blue and positive values in red. The more saturated the color in a cell, the higher is the magnitude of the DWT coefficient associated with that cell. Frequency bands of the DWT are shown in increasing order from bottom to top (V8: 0–9.76 Hz, W8: 9.76–19.5 Hz, W7: 19.5–39.1 Hz, W6: 39.1–78.1 Hz, W5: 78.1–156 Hz)

The solution can be found using the RoLDSIS technique. The 128 coefficients of vectors ${\mathbf {b}}_\Phi$ and ${\mathbf {b}}_\Psi$, called RoLDSIS *loadings*, can be represented in the form of a scalogram, which is a time-frequency representation, similar to the one used in [24]. This is depicted in Fig. 4. Notice that the representation space for ${\mathbf {b}}$ is identical to that for ${\mathbf {x}}$, i.e. the ${\mathbb {R}}^N$ feature space. In the scalograms, the magnitude of each coefficient is encoded by the color saturation, such that the paler the color, the closer the coefficient is to zero. The sign of the coefficient is encoded by the color, red and blue meaning negative and positive values, respectively. The vectors ${\mathbf {b}}$ can then be transformed into the time domain using THE inverse DWT. The associated time profiles for ${\mathbf {b}}_\Phi$ and ${\mathbf {b}}_\Psi$ are shown on the top of the respective scalograms in Fig. 4.

#### Projections onto physical and psychophysical directions

**Fig. 5:**
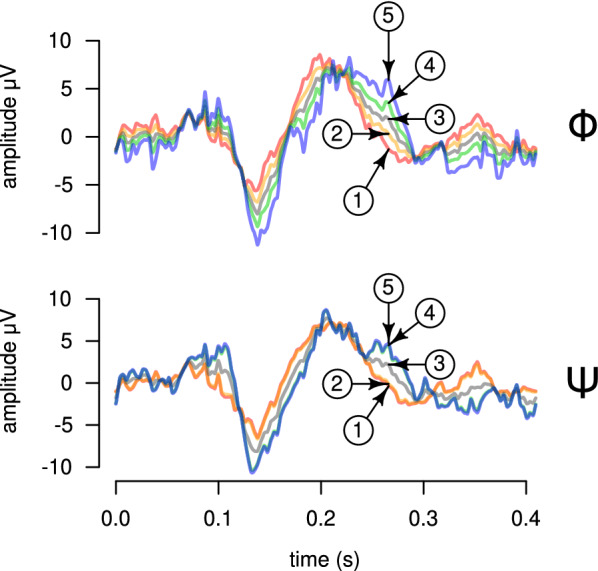
Projections of ERPs for stimuli #1 to #5 onto the axes found by RoLDSIS. The results shown are for a representative participant (the same as in Fig. 2. The responses projected onto the physical ($\Phi$) and psychophysical ($\Psi$) axes are shown in the top and bottom panels, respectively. Each projection, represented in the time domain, is drawn with a different color and indicated by the corresponding stimulus number. Note that, for the psychophysical case, the signals for stimuli #1 and #2, and for stimuli #4 and #5 are almost identical

The vectors ${\mathbf {b}}_\Phi$ and ${\mathbf {b}}_\Psi$ obtained by the RoLDSIS procedure can be interpreted as specific directions in the feature space. These directions would then indicate a sort of “canonical” representation of the neuronal activity that is associated with the variation in the stimulus attribute, either physical or psychophysical. The varying response along these directions can be represented in the time domain as in Fig. 5. Each curve in the figure is obtained by projecting the original observations ${\mathbf {x}}_i$ onto the respective direction and by using the inverse DWT to obtain the associated time profile.

#### Relationship between ${\mathbf {b}}_\Phi$–${\mathbf {b}}_\Psi$ divergence and the degree of categorization

In order to assess the relevance of the results obtained by the RoLDSIS technique, we computed the angle between the obtained physical and psychophysical directions. The value of this angle is specific to each participant and represents the separation between the neuronal representations of the two attributes. This angle can vary between 0$^\circ$ and 90$^\circ$, where a 0$^\circ$ angle means the physical and psychophysical representations are indistinguishable from each other whereas a 90$^\circ$ angle means they are uncorrelated (orthogonal) to each other. We investigated the relationship between this angle and the degree of categorization, which corresponds to the maximal slope of the psychometric curve fitted to the participant’s responses in the identification task (see Fig. 2).

**Fig. 6:**
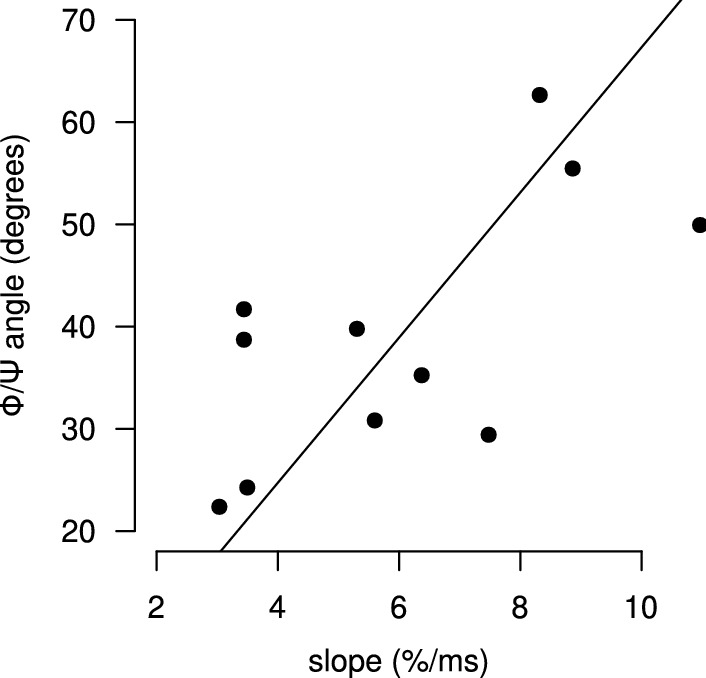
Relationship between the slope of the psychometric curve and the angle between the neurophysiological axes. In this population scatter plot, each point represents a participant. The horizontal and vertical axes represent, respectively, the slope of the fitted psychometric curve at 50% and the angle between the physical and the psychophysical directions obtained by the RoLDSIS procedure. The black line corresponds to the correlation line

The psychometric curve is described by the sigmoid function

19 $$\begin{aligned} p(t)=100/[1 + e^{\beta (t-t_0)}], \end{aligned}$$

where *t* is the VOT, *p*(*t*) is the probability of choosing /ta/ when VOT ${}=t$, and $t_0$ corresponds to the value of *t* at the curve’s inflection point ($p=50\%$). The maximal slope of the psychometric curve happens at $t=t_0$ and is equal to $(100/4)\beta$ (in %/ms units). A large value of $\beta$ indicates a stronger categorical perception by the participant [29]. The results for the 11 participants are shown in Fig. 6. As it can be seen in the figure, the angle is significantly correlated with the slope in the population (Pearson’s $r = 0.67$, $t[9] = 2.68$, $p < 0.05$).

### Assessment of the RoLDSIS technique

#### What if the regression problem were overdetermined?

**Fig. 7:**
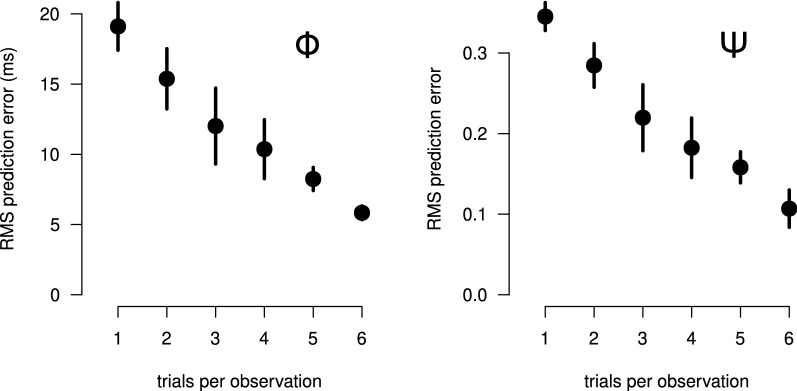
Prediction error of linear regression for overdetermined cases. Traditional least squares regression applied to the linear model relating ERP feature vectors and either physical (left) or psychophysical (right) attributes. The RMS prediction error is shown in the vertical axis. The number of trials per observation, varying from 1 to 6, is shown in the horizontal axis. Dots and vertical bars represent, respectively, the means and standard deviations obtained for the 11 participants

In our experiment, we computed the average of the ERPs for each stimulus, in order to reduce the SNR of the obtained signals. This results in an HDLSS problem, which usually requires the use of regularization to solve the regression problems defined by Eqs. 17 and 18 . The HDLSS problem can be potentially alleviated by having more data observations available. This can be done artificially, without actually collecting more data, by splitting the currently available data into groups containing a smaller number of trials. If the number of observations is larger than the number of features, the equation systems become overdetermined and classical least-squares linear regression can be used. We assessed this possibility by solving the regression with different numbers of trials per observation. We did it for all participants. For a number of trials per observation greater than one, the trials were assigned at random to each observation. The results for the root mean square (RMS) regression errors for the ${\mathbf {b}}_\Phi$ and the ${\mathbf {b}}_\Psi$ axes across the population are summarized in Fig. 7. The *y* attributes of our stimuli vary between − 52 and + 16 ms, in the $\phi$ case, and between 0 and 1, in the $\psi$ case. From the figure, one can see that the RMS regression errors are considerably high when there is one trial per observation, with the population mean being 19.1 ms in the $\phi$ case and 0.35 in the $\psi$ case. When the number of trials per observation increases, the RMS decreases almost linearly towards zero, a value that is theoretically attained when the number of observations is less than 128.

#### Reliability of the neurophysiological axes computation

**Fig. 8:**
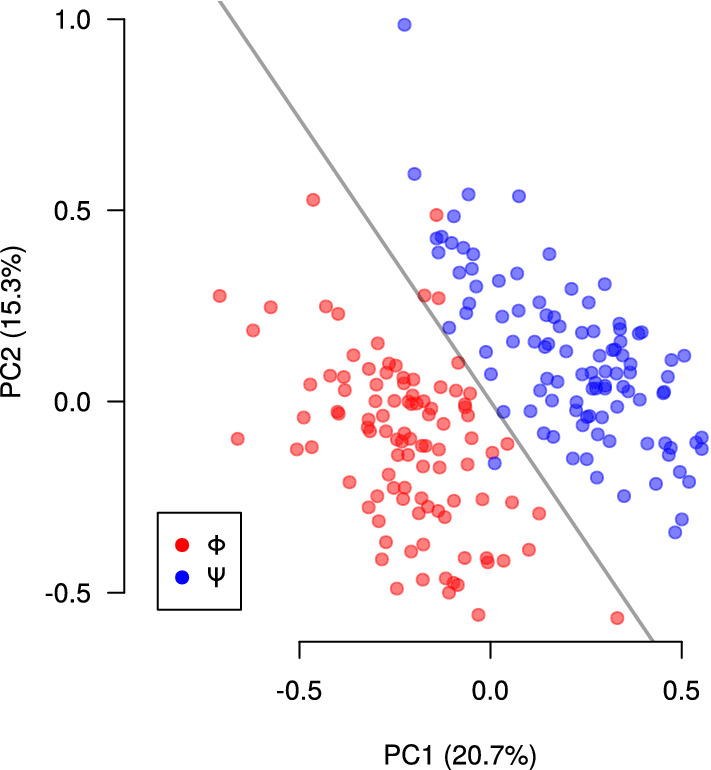
Bootstrap results of the RoLDSIS procedure. The samples obtained by the bootstrap procedure on the RoLDSIS for both physical ($\Phi$) and psychophysical ($\Psi$) are shown in red and blue dots, respectively, for a representative participant (the same as in Fig. 2). The horizontal and the vertical axes represent the first and second components of the PCA applied to RoLDSIS direction axis transformed into spherical coordinates. The percentage of variance explained by this two PCs are indicated in the axes labels. The gray line corresponds to the LDA separatrix

The separation between the ${\mathbf {b}}_\Phi$ and ${\mathbf {b}}_\Psi$ axes, expressed as the angle between these two directions (see Fig. 6) could be simply the result of a statistical fluke. In order to assess this issue, we ran a bootstrap procedure in which, for each participant and for each stimulus, the trials were resampled with replacement. One hundred new estimations for each ${\mathbf {b}}_\Phi$ axis and ${\mathbf {b}}_\Psi$ axis were thus obtained using RoLDSIS on each resampled data set. The values obtained in this procedure represent directions in the ${\mathbb {R}}^{128}$ space of wavelet features. The axes are unit vectors, lying on a 127-dimensional hypersphere, and can thus be represented by 127 spherical coordinates (the analogous of azimuth and elevation in a 3D sphere) [30]. In order to assess the results, PCA was applied to the set of 200 points (including both physical and psychophysical cases) transformed into spherical coordinates, using the prcomp function of the R software. This procedure was applied separately to each participant. The two first principal components (PCs) explain, on average, 50% of the variance, with a minimum of 32% and a maximum of 89% in the population. Linear discriminant analysis (LDA) was then applied to the data projected onto the first two PCs, using the lda function of MASS package of the R software [31]. LDA works by finding the linear transformation that maximizes the ratio between the inter-class and the intra-class variances. The resulting LDA separatrix defines the linear decision boundary that optimally separates the ${\mathbf {b}}_\Phi$ and the ${\mathbf {b}}_\Psi$ points. The reliability of the RoLDSIS procedure is assessed by the amount of LDA misclassifications, whose median value across participants is 7 (minimum value 0, maximum value 63). Figure 8 shows the results of this PCA–LDA procedure for a representative participant.

#### Comparison with regularized linear regression procedures

**Fig. 9:**
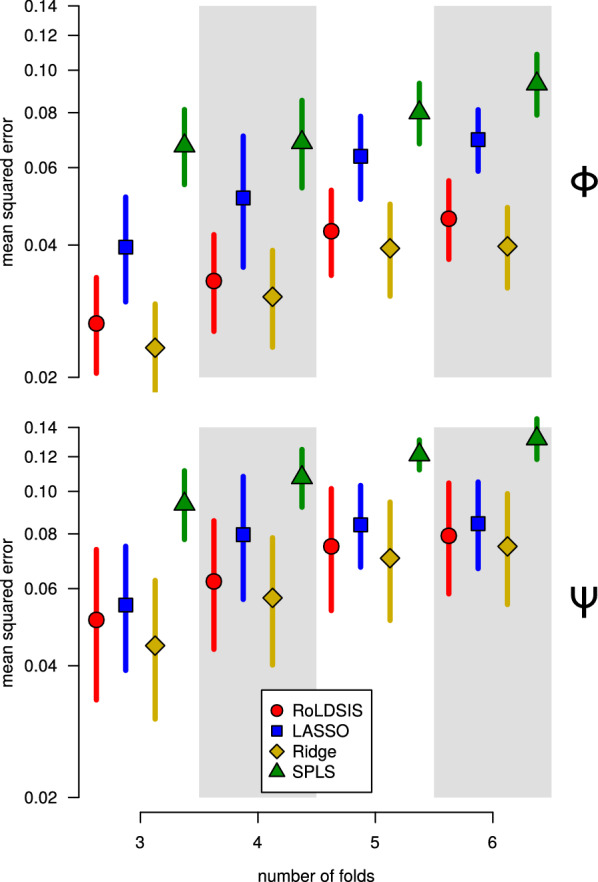
CV errors for the proposed regression method (RoLDSIS) and the methods of regularized linear regression. The results for the physical ($\Phi$) and the psychophysical ($\Psi$) attributes are shown in the top and the bottom panels, respectively. The mean squared errors for the test set of the CV are shown with dots. Confidence intervals at 95% are represented by vertical bars

A legitimate question that may arise at this point is how the RoLDSIS procedure compares with other regression techniques commonly used in HDLSS problems. In order to make this comparison, we considered three popular regression techniques, namely LASSO [32], Ridge Regression [9] and SPLS [10]. These techniques have regularization parameters ($\lambda$ for LASSO and Ridge Regression, and $\zeta$ and *K* for SPLS) whose optimal values can be found by using a CV procedure. This procedure runs as follows. First, the trials for each stimulus are randomly split into *k* groups, called *folds*. Second, we take each group in turn, put it aside as the test data set, and use the data in the remaining $k-1$ groups to fit the model. The fitted model is used to compute the prediction errors on the test data set. The total CV error is the mean value of the prediction errors computed across the *k* steps. The model fitting is done for specific values of the regularization parameters, according to the particular regression technique being tested. Using a gradient-descent optimization procedure, we found the optimal values of the regularization parameters that yield the minimum value of the CV error. Since RoLDSIS has no regularization parameter, the optimization procedure described above does not apply to it. This procedure was applied to each of the eleven participants and to each of the physical and psychophysical attributes. Figure 9 shows the population mean of the CV errors for the number of folds varying from 3 to 6, as well as the 95% confidence intervals of the mean estimations.

In order to assess how differently the regression techniques perform on our data, we fitted a linear mixed model to the results, considering the number of folds as a continuous fixed factor, the regression technique as a fixed discrete factor, and the participant as a random factor. The mean squared error (MSE) values, which follow a $\chi ^2$ distribution, were transformed to normal [33] and the resulting values were used as the dependent variable of the linear model. The results show a significant increase in MSE with the number of folds ($F[1,158]=50.4, p < 0.001$ for physical and $F[1,158]=32.2, p < 0.001$ for psychophysical). For the physical case, there was a significant effect for the method factor ($F[3,158]=5.22, p < 0.01$), and multiple comparisons showed significant differences among all pairs of methods, besides the RoLDSIS / Ridge Regression pair. For the physical case, the method factor has a marginal effect ($F[3,158]=2.47, p < 0.064$). In this later case, no significant differences were found between RoLDSIS, LASSO, and Ridge Regression, but SPLS was significantly different from the others.

#### Time-frequency representation of the neurophysiological axes

As illustrated in Fig. 4, the RoLDSIS results can be useful for revealing the locations, in the time-frequency domain, associated with the stimulus attributes (physical and psychophysical in the present paper). Since the regression is obtained on an individual basis, the patterns of time-frequency distribution associated with the neurophysiological axis may differ from one participant to another. Therefore, it would be interesting to know whether there are global time-frequency patterns that arise in the population.

This investigation involved RoLDSIS, as well as the three other regression techniques considered in the previous section, and consisted in the computation of the population-wide histogram of the neurophysiological axis in the time-frequency domain. The first step of this analysis is to compute the squared values of the components of the axis ${\mathbf {b}}$ obtained by the regression technique. The squared value of a given wavelet coefficient can be interpreted as the importance (or the “energy”) of the neurophysiological axis at the associated time-frequency slot. The resulting values were then accumulated for all participants, separately for the ${\mathbf {b}}_\Phi$ and the ${\mathbf {b}}_\Psi$ axes, and the square root was computed for the sums at each wavelet component. For the RoLDSIS technique, the average of the ERPs for each stimulus were used for doing the regression, whereas, for the other techniques, the regression result of the 3-fold CV were used (see previous section).

**Fig. 10:**
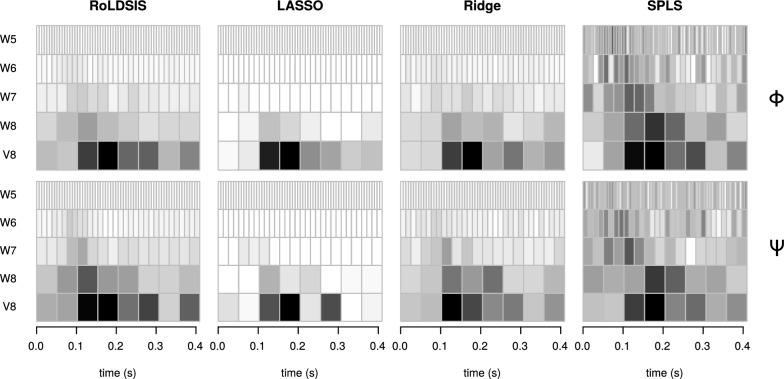
Scalograms of the regression results. Scalograms for the root mean squared regression coefficients for each component of the DWT, across the population, are shown for the proposed regression method (RoLDSIS), for the methods of regularized linear regression. The results for the physical ($\Phi$) and the psychophysical ($\Psi$) attributes are shown in the top and the bottom panels, respectively. Shades of gray represent the cumulative RMS (white for zero and black for the maximum value). Frequency bands of the DWT are the same as those in Fig. 4

The results are shown in Fig. 10 in the form of time-frequency scalograms. The darker a DWT component appears in a scalogram, the more it contributes to the associated neurophysiological direction across the population.

## Discussion

In this paper, we evaluated and presented a new regression technique, called RoLDSIS, to deal with the HDLSS problem in ERP processing, which is a special case of dimension reduction [11]. The RoLDSIS technique is based on the assumption that the solution of the regression problem relating the ERPs to attributes of the stimuli lies in the subspace spanned by the ERP observations ${\mathbf {x}}_i$, whose number *M* can be much smaller than the dimensionality of the feature space. This is a reasonable assumption in ERP studies, as the one presented in this paper. Indeed, since EEG signals are known to be highly redundant [34], the intrinsic dimensionality of realistic data sets should be lower than the dimension of the feature space. Under the assumption of low SNR, the *M* observations ${\mathbf {x}}_i$ must be contained in this subspace of lower dimensionality. Therefore, in the absence of further information about this subspace, and supposing that the available *M* observations are reliable measurement of the true ERPs, we restrict the analysis to the space spanned by these observations. Below we discuss some aspects of the evaluation of the RoLDSIS technique applied to the study presented in this paper.

### RoLDSIS is suitable when grand averaging is needed

SNR is typically poor in EEG and hundreds of trials are needed for obtaining a single ERP observation. The estimated SNR of the raw signals in our experiment is between −12 dB and −15 dB. These values are extremely low for doing a trial-by-trial analysis. In order to obtain ERPs with acceptable SNR levels, we compute the grand average of the trials for each participant and each stimulus. In the ideal case where 200 trials per stimulus are used, the SNR increases by $10 \log _{10}{200} = 23$ dB, yielding a final SNR between 8 dB and 11 dB. Since this is a reasonable level, we limited the total number of trials to 1000 (5 stimuli, 200 trials per stimulus). The duration of the EEG acquisition was thus 35 minutes per participant. Doing experiments longer than that could result in undesirable fatigue-related effects.

As we have seen in the Section “*What if the regression problem was overdetermined?*”, using ERPs with high SNR is crucial for obtaining reliable results in the regression procedure. The best possible SNR level is obviously obtained when all the data for a given stimulus is used for computing the grand-averaged ERP. This prevents the use of classic regularization techniques, such as LASSO, Ridge Regression or SPLS, since these techniques have free regularization parameters whose optimal values require the use of a CV procedure to be determined. In reduction dimension techniques, such as SPLS, the number of dimensions in the reduced space is also a free parameter and must therefore be obtained by CV. As we demonstrated in Section “*Comparison with regularized linear regression procedures*”, CV produces adequate results only if more observations are obtained from the data, what can dramatically decreases the SNR and degrade the regression results. RoLDSIS does not have this problem, since it does not contain regularization parameters and assumes that the reduced subspace is the one spanned by the available observations.

### Separation between the ${\mathbf {b}}_\Phi$ and ${\mathbf {b}}_\Psi$ axes

We applied RoLDSIS to the problem of phonemic categorization, where both physical (VOT) and psychophysical (probability of syllable identification) attributes were associated with the ERP observations. This was mathematically formulated as a linear regression problem whose solutions are vectors (${\mathbf {b}}_\Phi$ for the physical attribute and ${\mathbf {b}}_\Psi$ for the psychophysical attribute). These vectors define axes in the subspace spanned by the observations.

In the Section “*Reliability of the attribute axes computation*”, we showed that our data allows the computation of ${\mathbf {b}}_\Phi$ and ${\mathbf {b}}_\Psi$ axes that are significantly different from each other. This demonstrates the suitability of RoLDSIS for finding the neurophysiological correlates of the physical and psychophysical processes in speech perception. Furthermore, the RoLDSIS results, obtained from the electrophysiological data, can be directly associated with the behavioral results. Indeed, as we showed in the Section *“Results”*, there is a significant correlation between the angle separating the ${\mathbf {b}}_\Phi$ and ${\mathbf {b}}_\Psi$ axes and the degree of categorization of the participant, which is expressed by the maximal slope of the psychometric curve. Our findings suggest that participants who present stronger categorization (i.e. whose psychometric curve has a higher slope) have more distinguishable physical and psychophysical representations of the phonemes.

We note that previous studies, like the ones by Bidelman and colleagues [16, 29], have already tried to associate the degree of categorization with neurophysiological features extracted from ERP signals. However, to our knowledge, the study in the present paper is the first one that attempts to associate stimulus attributes with the whole set of extracted features (thanks to the regression technique) without any *a priori* definition of the neurophysiological correlates.

### On the time-frequency characteristics of the neurophysiological axes

As can be observed in Fig. 5, the signals resulting from the projections on a given neurophysiological axis reflect the values of the attribute associated with that axis. For instance, for the ${\mathbf {b}}_\Psi$ axis, projections of stimuli #1 and #2 are almost indistinguishable. This also happens with stimuli #4 and #5. This mimics the values of the $\psi$ attribute, which are 0.0, 0.05, 0.5, 0.95, and 1.0. An equivalent result can be observed for projections on the ${\mathbf {b}}_\Phi$ axis, where stimuli #2 and #4 are closer to stimulus #3 than to #1 and #5, respectively. This mimics the values of the VOT of those stimuli (see the abscissa of the plot in Fig. 2), which are the values of the $\phi$ attribute.

Another interesting observation concerning the projections on the neurophysiological axes is that the separation between the projections stimuli #1 and #5 varies over time. This variation is typically different between the ${\mathbf {b}}_\Phi$ and ${\mathbf {b}}_\Psi$ axes. For instance, in the data shown in Fig. 5, the projections of the five stimuli collapse to the same value around $t =$180 ms for ${\mathbf {b}}_\Phi$, while stimuli #1 and #5 are well apart at that time for ${\mathbf {b}}_\Psi$. A more precise analysis of the differences between the ${\mathbf {b}}_\Phi$ and ${\mathbf {b}}_\Psi$ axes can be found by inspecting the scalograms representing them (Fig. 4). Indeed, we can see that the effects described above are due to the wavelet coefficients in bands V8 and W7 around $t =$180 ms. These wavelet coefficients have stronger loadings for the ${\mathbf {b}}_\Phi$ axis, in comparison with the ${\mathbf {b}}_\Psi$ axis.

These differences in the loadings of the ${\mathbf {b}}_\Phi$ and ${\mathbf {b}}_\Psi$ axes indicate different neurophysiological representations for the stimuli attributes. In our data, we observed that the RoLDSIS loadings are participant-specific, what indicates idiosyncratic ways of VOT processing and phonemic categorization. However, at the population level, the loadings are concentrated at specific regions of the time-frequency domain (see Fig. 10). Our results are compatible with evidence reported elsewhere [15–17, 35], in terms of neurophysiological correlates of phonemic categorization. For instance, Bouton and colleagues [17] observed that the tracking of a specific acoustic cue happens in the time interval 95–120 ms and again around 175 ms. Chang and colleagues [15] showed that maximum consonant categorization happens in the superior temporal gyrus (STG) at around 110 ms. Also, previous studies show the importance of $\uptheta$ oscillations (our V8 DWT band), $\upbeta$ oscillations (W8 and W7 bands) and low-$\upgamma$ oscillations (W6 band) in speech processing [27, 28], what is also shown in our results. In sum, these findings corroborate the usefulness of RoLDSIS and open a new avenue in the identification of neurophysiological correlates of speech perception.

### Advantages and limitations of RoLDSIS in relation to regularized regression techniques

Regression techniques are used for estimating the functional relationship between the pairs of observations/attributes $\{{\mathbf {x}}_i,y_i\}$, in the form $y=f({\mathbf {x}}_i)$ or, as in the case of this paper, $y = a+{\mathbf {b}}^\intercal {\mathbf {x}}$. There are two main reasons for estimating *f*, namely for doing *prediction* and for doing *inference* [11]. RoLDSIS is clearly more adapted for doing the later rather than the former. Indeed, prediction would not make sense in experiments like the one presented in this paper, since the number of observations per participant is extremely small and there is no extra data on which the predictive power of the inferred neurophysiological axis ${\mathbf {b}}$ could be tested. In contrast, RoLDSIS seems to be useful for identifying the neurophysiological correlates of perceptual processes and to determine how these correlates are expressed in terms of time/frequency features. This could also be extended to the scalp topography, in the case where more sensors are used to measure the neuronal activity.

The RoLDSIS technique is a special case of principal component regression (PCR) [36] in which the maximum number of principal components is used, namely $M-1$, where *M* is the number of observations. Thanks to this, the solution of the linear regression is exact, meaning that the regression error is equal to zero. In other words, RoLDSIS pushes PCR to the limit, while avoiding an overdetermined system of equations, which would happen if the number of unknowns were greater than *M*. As such, RoLDSIS does not suffer from the problem of feature selection faced by PCR. Indeed, in regular PCR, there is no guarantee that the first PCs will be associated with the attribute *y* in a meaningful way. RoLDSIS assumes that the neurophysiological axis (represented by ${\mathbf {b}}$) is contained into the subspace spanned by the observations. This is a reasonable assumption, provided that the SNR is high, meaning that the observations are a reliable representation of the underlying neurophysiological mechanism that produce the ERPs. It should be noted that, in the case of a large amount of reliable (i.e. with high SNR) observations, a CV analysis with PCR can be performed in order to determine the optimal number of PCs. This optimal dimension may be inferior to $M-1$, which is the value used by RoLDSIS.

In the CV analysis, we showed that RoLDSIS and Ridge Regression perform similarly on our data set, in terms of CV errors (Fig. 9). Furthermore, there is evidence that RoLDSIS performs better than LASSO and SPLS. Interestingly enough, RoLDSIS and Ridge Regression yield similar time-frequency representations for the neurophysiological axes, at least at the population level (Fig. 10). The LASSO technique concentrates the regression loadings on a set of features. On the other hand, the SPLS technique shows overdispersion in the loadings distribution, limiting the interpretation power of its results. We can therefore conclude that RoLDSIS yields results closer to those of a *parameter shrinkage* method (like Ridge Regression) rather than to those of a *parameter selection* method (like LASSO or SPLS) [11]. The similarity between the RoLDSIS and Ridge Regression results is somehow surprising because, as we presented in the Section “*Background*”, RoLDSIS is a special case of PCR. At any rate, RoLDSIS has an advantage with respect to regularized regression techniques, namely the absence of regularization parameters and the ability of producing analytical results without the need for CV procedures.

Finally, we note that RoLDSIS makes two basic assumptions related to linearity. First, we restrict the neurophysiological axis to be contained in the linear subspace of the feature space ${\mathbb {R}}^N$ spanned by the *M* observations. Instead of this, it is possible to find a non-linear manifold that contains the observations and that has a dimension smaller than $M-1$. That would imply the inclusion of extra free parameters to describe that manifold and the parameter-free aspect of RoLDSIS would be lost. The second assumption is that, once the subspace is determined, we hypothesize a linear relationship between the observations and the stimuli attributes (Eq. 1). Notice that there is no advantage to suppose a more complex relationship than the linear, since the number of unknowns in the linear system is exactly equal to the number of observations.

## Conclusion

In this paper, we proposed a regression technique, called RoLDSIS, that addresses the HDLSS problem in EEG data sets, where hundreds of features are extracted from the ERP signals and the number observations is very limited. Many popular regularized regression techniques exist that tackle this problem. However, these techniques require the specification of regularization parameters and, consequently, a relatively high number of observations in order to run reliable CV procedures. In contrast, RoLDSIS assumes that the regression solution is embedded in the subspace spanned by the observations. This allows the regression problem to be solved exactly, even when the number of observations is extremely small. In particular, this technique may be useful for EEG experiments, where ERPs must be averaged over many repetitions of a small number of presented stimulus in order to improve the SNR. We applied RoLDSIS to the analysis of data from an EEG experiment that aimed to find the neurophysiological correlates of phonemic categorization. The results obtained by regressing the wavelet-transformed ERPs against the physical and psychophysical attributes of the stimuli showed relevant characteristics of speech categorical perception in the time-frequency domain. In our data set, the prediction errors obtained by RoLDSIS are comparable to those obtained with Ridge Regression and smaller than those obtained with LASSO and SPLS. In conclusion, even though RoLDSIS is a simple technique, it is suitable for the processing and interpretation of neurophysiological signals.

## Data Availability

The data that support the findings of this study, as well as the scripts for reproducing the results, are available in the repository https://github.com/RoLDSIS/code.

## Abbreviations

ABR: Auditory brainstem response
BCI: Brain-computer interface
CV: Cross-validation
DWT: Discrete wavelet transform
ECoG: Electrocorticography
ERP: Event-related potential
EEG: Electroencephalography
fMRI: Functional magnetic resonance imaging
HDLSS: High dimension, low sample size
LASSO: Least absolute shrinkage and selection operator
LDA: Linear discriminant analysis
MEG: Magnetoencephalography
MSE: Mean squared error
NIRS: Near infrared spectroscopy
PCs: Principal components
PCA: Principal component analysis
PCR: Principal component regression
RMS: Root mean square
RoLDSIS: Regression on low-dimension spanned input space
SNR: Signal-to-noise ratio
SPLS: Sparse partial least squares
STG: Superior temporal gyrus
VOT: Voice onset time
